# Inanspruchnahme zahnmedizinischer Versorgung von Erwachsenen mit und ohne Beeinträchtigungen und Behinderungen – Ergebnisse der Studie GEDA 2014/2015-EHIS

**DOI:** 10.1007/s00103-023-03748-7

**Published:** 2023-07-14

**Authors:** Laura Krause, Peter Schmidt, Stefanie Seeling, Franziska Prütz

**Affiliations:** 1 Abteilung für Epidemiologie und Gesundheitsmonitoring, Robert Koch-Institut, General-Pape-Str. 62–66, 12101 Berlin, Deutschland; 2 Abteilung für Behindertenorientierte Zahnmedizin, Universität Witten/Herdecke, Witten, Deutschland

**Keywords:** Mundgesundheit, Gesundheitliche Einschränkung, Zahnärztliche Praxis, Gesundheitsversorgung, Gesundheitsmonitoring, Oral health, Health limitation, Dental practice, Healthcare, Health monitoring

## Abstract

**Hintergrund:**

Beeinträchtigungen und Behinderungen können sich nachteilig auf die Mundgesundheit auswirken. Studien zur Mundgesundheit von Menschen mit Beeinträchtigungen und Behinderungen gibt es jedoch nur wenige. Dieser Beitrag untersucht die 12-Monats-Prävalenz der Inanspruchnahme zahnmedizinischer Versorgung von Erwachsenen mit und ohne Beeinträchtigungen und Behinderungen in Deutschland.

**Methoden:**

Die Analysen basieren auf Daten von 23.372 Personen ab 18 Jahren mit ständigem Wohnsitz in Deutschland der Studie GEDA 2014/2015-EHIS. Die Teilnehmenden wurden gefragt, wann sie zuletzt bei einem Zahnarzt, Kieferorthopäden oder einem anderen zahnmedizinischen Spezialisten waren, um sich selbst beraten, untersuchen oder behandeln zu lassen – „vor weniger als 6 Monaten“, „vor 6 bis weniger als 12 Monaten“, „vor 12 Monaten oder länger“ oder „nie“. Für die Analysen wurden die ersten und letzten beiden Antwortoptionen zusammengefasst, um die 12-Monats-Prävalenz der Inanspruchnahme zahnmedizinischer Leistungen zu erhalten.

**Ergebnisse:**

Erwachsene mit Beeinträchtigungen und Behinderungen haben im Jahr vor der Befragung etwas häufiger keine zahnmedizinische Praxis aufgesucht als Erwachsene ohne Beeinträchtigungen und Behinderungen (21,5 % vs. 18,4 %; *p* = 0,002). Der Zusammenhang zwischen dem Vorliegen von Beeinträchtigungen und Behinderungen und einer geringeren Inanspruchnahme zahnmedizinischer Versorgung blieb aber nach Kontrolle für Alter, Geschlecht, Partnerschaft und sozioökonomischen Status nicht bestehen.

**Diskussion:**

Es zeigen sich kaum Unterschiede in der Inanspruchnahme zahnmedizinischer Leistungen zwischen Personen mit und ohne Beeinträchtigungen und Behinderungen. Dennoch ist aufgrund ihrer im Mittel schlechteren Mundgesundheit zu überlegen, wie die zahnmedizinische Versorgung dieser sehr heterogenen Gruppe weiter verbessert werden kann. Die Auswertungen zeigen Versorgungsbedarfe und Präventionspotenziale auf.

## Abstract (en)

**Background:**

Impairments and disabilities can have a negative impact on oral health. However, studies on the oral health of people with impairments and disabilities are rare. This article examines the 12-month prevalence of dental utilization among adults with and without impairments and disabilities in Germany.

**Methods:**

Analyses are based on data from 23,372 persons aged 18 years and older with permanent residency in Germany from the GEDA 2014/2015-EHIS study. Participants were asked when they last visited a dentist or orthodontist on their own behalf – “less than 6 months ago,” “6 to less than 12 months ago,” “12 months ago or longer,” or “never.” For the analyses, the first two and the last two response options were combined, giving the 12-month prevalence of dental utilization.

**Results:**

Adults with impairments and disabilities were slightly more likely not to have visited a dental practice in the year prior to the survey than adults without impairments and disabilities (21.5% and 18.4%, respectively; *p* = 0.002). However, the association between the presence of impairments and disabilities and lower dental utilization did not persist after controlling for age, gender, partnership, and socioeconomic status.

**Discussion:**

There are hardly any differences in the dental utilization between persons with and without impairments and disabilities. However, due to their poorer oral health on average, it is necessary to consider how the dental care of this very heterogeneous group can be further improved. The analyses point to the need for care and prevention potentials.

## Einleitung

Gesundheitliche Beeinträchtigungen und Behinderungen betreffen einen großen Teil der Bevölkerung in Deutschland. In Zahlen betrachtet lebten im Jahr 2019 in Deutschland 10,4 Mio. Menschen mit einer amtlich anerkannten Behinderung in Privathaushalten. Dies entspricht 12,7 % der in Privathaushalten lebenden Einwohnerinnen und Einwohner [1]. Eine amtlich anerkannte Schwerbehinderung, d. h. einen Grad der Behinderung (GdB) von 50 oder höher, hatten im Jahr 2021 9,4 % der Menschen in Deutschland [2]. Weitaus größer ist der Anteil von Menschen mit Beeinträchtigungen, der auf fast 16,0 % der Bevölkerung geschätzt wird [3].

Menschen mit Beeinträchtigungen und Behinderungen sind eine heterogene Gruppe, in der ganz unterschiedliche gesundheitliche Situationen und Bedarfe existieren. Die Infobox enthält Definitionen von Beeinträchtigung, Behinderung und Schwerbehinderung, die dem Dritten Teilhabebericht der Bundesregierung [3] entnommen wurden. Basierend auf dem Artikel 25 der Behindertenrechtskonvention der Vereinten Nationen (UN-BRK) [4] haben all diese Personen das Recht „auf das erreichbare Höchstmaß an Gesundheit ohne Diskriminierung aufgrund von Behinderung“ [5]. Deutschland hat die UN-BRK [4] bereits im Jahr 2009 ratifiziert, also vor mehr als 10 Jahren. Dadurch wurde auch der Artikel 31 rechtsgültig, der die Verpflichtung zur „Sammlung geeigneter Informationen, einschließlich statistischer Angaben und Forschungsdaten“ [5], enthält. Zur gesundheitlichen Lage von Menschen mit Beeinträchtigungen und Behinderungen gibt es bislang aber nur wenige Daten, die auf bundesweiten, repräsentativen Erhebungen basieren. Dazu gehören die Gesundheitssurveys des Robert Koch-Instituts (RKI).

### Infobox Menschen mit Beeinträchtigungen und Behinderungen – Definitionen aus dem Dritten Teilhabebericht

Quelle: Bundesministerium für Arbeit und Soziales (2021; [3])

1. **Menschen mit Beeinträchtigungen**

Als solche werden Menschen bezeichnet, die im Zusammenhang mit Schädigungen von Körperstrukturen und -funktionen dauerhaft bei Aktivitäten beeinträchtigt sind. Menschen mit Beeinträchtigungen werden je nach Datenquelle statistisch unterschiedlich abgegrenzt. Gemeinsam ist allen so bezeichneten Gruppen jedoch, dass die zugehörigen Personen durch ihre Beeinträchtigungen nicht unbedingt bei ihren Aktivitäten im Alltagsleben eingeschränkt sein müssen, sie können es aber gleichwohl sein.

2. **Menschen mit Behinderungen**

Hierbei handelt es sich um Menschen, die bei Aktivitäten im Alltagsleben und/oder bei der gleichberechtigten Teilhabe durch Wechselwirkungen von eigenen Beeinträchtigungen und Barrieren in der Umwelt behindert werden. Dabei spielt es keine Rolle, ob es sich um eine amtlich anerkannte Behinderung oder Schwerbehinderung handelt.

3. **Menschen mit anerkannter Behinderung und anerkannter Schwerbehinderung**

Zu den Menschen mit einer anerkannten Behinderung oder einer anerkannten Schwerbehinderung zählen alle Personen, deren Behinderung von einem zuständigen Amt festgestellt beziehungsweise anerkannt wurde. Mit der Anerkennung geht die Vergabe eines Schweregrades der Behinderung in Form eines Grades der Behinderung (GdB) einher. Wenn ein GdB von 50 oder mehr vergeben wurde, so hat diese Person eine anerkannte Schwerbehinderung.

Das Vorliegen von Beeinträchtigungen und Behinderungen kann sich nachteilig auf die Mundgesundheit und die Inanspruchnahme zahnmedizinischer Leistungen auswirken [6]. Sowohl geistige und psychische Behinderungen als auch Seh- und Hörbehinderungen sowie körperliche Behinderungen (z. B. beim Greifen) gehen häufig mit einer schlechteren Mundgesundheit einher [6]. Die Anzahl an Studien zur Mundgesundheit von Erwachsenen mit Behinderungen in Deutschland ist jedoch sehr gering [7–10]. Die wenigen verfügbaren Studien untersuchen zudem vorrangig die Mundgesundheit von Erwachsenen mit geistiger Behinderung [7–10]. Die Ergebnisse zeigen, dass bei jungen Erwachsenen mit geistiger Behinderung die mittlere Karieserfahrung auf vergleichbarem Niveau lag wie bei jungen Erwachsenen ohne geistige Behinderung. Jedoch wiesen sie im Mittel mehr fehlende Zähne (höhere MT[„missing teeth“]-Komponente) auf [6]. Auch lang andauernde gesundheitliche Beeinträchtigungen können die Mundgesundheit negativ beeinflussen [11]. Umgekehrt kann eine unzureichende Mundgesundheit das Auftreten verschiedener Allgemeinerkrankungen begünstigen. Studien zeigen dies z. B. für Diabetes, Herz-Kreislauf- und Atemwegserkrankungen [12–14].

Die Inanspruchnahme zahnmedizinischer Leistungen wurde bislang nur für Erwachsene mit Behinderungen untersucht [10, 15, 16]. Mit den Daten des RKI-Gesundheitsmonitorings kann das Vorliegen von Beeinträchtigungen und Behinderungen kombiniert ausgewertet werden [17]. Vor diesem Hintergrund wird im Folgenden untersucht, ob sich Erwachsene mit und ohne Beeinträchtigungen und Behinderungen in der 12-Monats-Prävalenz der Inanspruchnahme zahnmedizinischer Versorgung unterscheiden. Dabei werden Unterschiede nach soziodemografischen Merkmalen wie Alter, Geschlecht, Partnerschaft und sozioökonomischem Status (SES) dargestellt.

## Methoden

### Stichprobendesign und Studiendurchführung

Gesundheit in Deutschland aktuell (GEDA) ist eine bundesweite Befragungsstudie des RKI zur gesundheitlichen Lage der erwachsenen Bevölkerung ab 18 Jahren. In GEDA 2014/2015 wurde erstmals vollständig der Fragebogen der Europäischen Gesundheitsumfrage (European Health Interview Survey, EHIS Wave 2) integriert [18]. Die Erhebung erfolgte mittels eines Fragebogens, der als Papier- oder Onlineversion ausgefüllt werden konnte [17]. GEDA 2014/2015-EHIS basiert auf einer 2‑stufig geschichteten Cluster-Stichprobe. Dafür wurden zunächst 301 Gemeinden nach dem Zufallsprinzip ausgewählt. Diese entfallen auf 231 Kreise und kreisfreie Städte und repräsentieren die verschiedenen Gemeindegrößen und Regionen in Deutschland. Die Auswahl erfolgte durch das GESIS – Leibniz-Institut für Sozialwissenschaften in Mannheim. Gemeinden, die weniger als 1000 Einwohnerinnen und Einwohner hatten, wurden mit ähnlichen kleinen Nachbargemeinden zu einem Auswahlort kombiniert. Mehrere Großstädte wurden aufgrund ihrer großen Bevölkerung durch mehrere Auswahlorte repräsentiert. Anschließend wurden Personen mit ständigem Wohnsitz in den ausgewählten Gemeinden zufällig aus den lokalen Bevölkerungsregistern gezogen. Personen, die in (teil-)stationären Wohnformen oder Einrichtungen lebten, wurden nicht befragt. Insgesamt haben 24.016 Personen (13.144 Frauen und 10.872 Männer) an der Studie teilgenommen (Response: 26,9 %).

### Inanspruchnahme zahnmedizinischer Versorgung

Zur Inanspruchnahme zahnmedizinischer Leistungen wurde in der Studie gefragt: „Wann waren Sie zuletzt bei einem Zahnarzt, Kieferorthopäden oder einem anderen zahnmedizinischen Spezialisten, um sich selbst beraten, untersuchen oder behandeln zu lassen?“ Antwortmöglichkeiten waren: „vor weniger als 6 Monaten“, „vor 6 bis weniger als 12 Monaten“, „vor 12 Monaten oder länger“ und „nie“. Für die statistischen Analysen wurden die ersten beiden sowie letzten beiden Antwortkategorien zusammengefasst, sodass die 12-Monats-Prävalenz der Inanspruchnahme zahnmedizinischer Leistungen (Ja/Nein) abgebildet werden kann [19].

### Beeinträchtigungen und Behinderungen

Die Variable zu Beeinträchtigungen und Behinderungen wurde für die Auswertungen wie im Zweiten Teilhabebericht [20] operationalisiert: Als Personen mit Beeinträchtigungen und Behinderungen werden alle Teilnehmenden verstanden, bei denen eine *amtlich anerkannte Schwerbehinderung (GdB* *≥* *50) oder eine starke, länger als 6 Monate andauernde krankheitsbedingte Einschränkung bei der Ausübung von Alltagstätigkeiten* besteht. Zum Vorliegen einer Behinderung wurden die Teilnehmenden gefragt: „Haben Sie eine Behinderung, die vom Versorgungsamt amtlich anerkannt ist?“, und, wenn mit „Ja“ geantwortet wurde: „Welcher Grad der Behinderung ist bei Ihnen amtlich anerkannt?“ Beeinträchtigungen wurden mit folgender Frage erfasst: „Sind Sie dauerhaft durch ein gesundheitliches Problem bei Tätigkeiten des normalen Alltagslebens eingeschränkt?“ Wurde diese Frage mit „Ja“ beantwortet, wurde anschließend nach der Stärke („Wie stark sind die Einschränkungen?“, mögliche Antworten: „stark eingeschränkt“, „mäßig eingeschränkt“) und der Dauer der Einschränkungen gefragt („Wie lange dauern Ihre Einschränkungen bereits an?“, mögliche Antworten: „weniger als 6 Monate“, „6 Monate und länger“ [21]).

### Soziodemografische Merkmale

Die Einteilung der Altersgruppen erfolgte in Anlehnung an den Zweiten Teilhabebericht der Bundesregierung in 18 bis 64 Jahre, 65 bis 79 Jahre sowie 80 Jahre und älter [20]. Ob eine Partnerschaft besteht, wurde anhand der Frage festgestellt: „Leben Sie mit einer Person aus Ihrem Haushalt in einer Ehe oder eheähnlichen Gemeinschaft?“ (Ja/Nein). Der SES beruht auf einem mehrdimensionalen additiven Index, in den Angaben der Teilnehmenden zum Bildungsstand, zur Einkommenssituation und zum Berufsstatus eingehen [22].

### Statistische Analysen

Die Auswertungen basieren auf Daten von 23.372 Personen (12.747 Frauen und 10.625 Männer) mit gültigen Angaben zu krankheitsbedingten dauerhaften Einschränkungen sowie amtlich anerkannter Schwerbehinderung (Tab. 1). Untersucht wird, ob sich Erwachsene mit und ohne Beeinträchtigungen und Behinderungen in der Inanspruchnahme zahnmedizinischer Leistungen unterscheiden. Dabei werden Unterschiede nach Geschlecht, Alter, Partnerschaft und SES berichtet. Ausgewiesen werden Prävalenzen sowie Prevalence Ratios (PR) und *p*-Werte aus univariaten und multivariaten log-Poisson-Regressionen. Die multivariaten Regressionsmodelle wurden statistisch kontrolliert für die genannten soziodemografischen Merkmale. Es wird von einem statistisch signifikanten Unterschied in der Inanspruchnahme zahnmedizinischer Versorgung zwischen Personen mit und ohne Beeinträchtigungen und Behinderungen ausgegangen, wenn der *p*-Wert kleiner als 0,05 ist. Durchgeführt wurden die Analysen mit einem Gewichtungsfaktor, der Abweichungen der Stichprobe von der Bevölkerungsstruktur (Stand: 31.12.2014) bezüglich Geschlecht, Alter, Kreistyp und Bildung korrigiert. Der Kreistyp spiegelt den Grad der Urbanisierung und entspricht der regionalen Verteilung in Deutschland. Die Internationale Standardklassifikation für das Bildungswesen (ISCED) wurde verwendet, um die schulischen und beruflichen Bildungsabschlüsse der Teilnehmenden zu gruppieren [23]. Alle Analysen wurden mit den Survey-Prozeduren von Stata 17.0 gerechnet (Stata Corp., College Station, TX, USA, 2015). Eine ausführliche methodische Darstellung von GEDA 2014/2015-EHIS ist andernorts publiziert [24, 25].

**Table 1:** 

	Fallzahl (*n*)	Ungewichtete Stichprobe (%)	Gewichtete Stichprobe (%)
*Beeinträchtigungen und Behinderungen*			
Ja	2911	12,5	13,5
Nein	20.461	87,5	86,5
*Geschlecht*			
Frauen	12.747	54,5	50,8
Männer	10.625	45,5	49,2
*Alter*			
18–64 Jahre	17.884	76,5	76,3
65–79 Jahre	4454	19,1	19,0
80 Jahre und älter	1034	4,4	4,8
*Partnerschaft*			
Ja	15.767	67,5	67,6
Nein	7312	31,3	32,4
Fehlende Werte	293	1,3	–
*Sozioökonomischer Status*			
Niedrig	3693	15,8	19,8
Mittel	13.086	56,0	60,0
Hoch	6544	28,0	20,2
Fehlende Werte	49	0,2	–
*Inanspruchnahme zahnmedizinischer Versorgung*			
Ja, in den letzten 12 Monaten	19.192	82,1	81,2
Nein	4080	17,5	18,8
Fehlende Werte	100	0,4	–

## Ergebnisse

Von den insgesamt 23.372 in die Analysen eingeschlossenen Personen gaben 2911 Befragte an, von Beeinträchtigungen und Behinderungen betroffen zu sein (Tab. 1). Davon hatten 2256 Personen eine amtlich anerkannte Schwerbehinderung (9,4 %, bezogen auf die gesamte Studienpopulation), bei 1406 Personen (5,9 %) bestand eine dauerhafte krankheitsbedingte Einschränkung. 751 Personen (3,1 %) nannten sowohl eine amtlich anerkannte Schwerbehinderung als auch eine krankheitsbedingte dauerhafte Einschränkung (Ergebnisse nicht gezeigt).

Erwachsene mit und ohne Beeinträchtigungen und Behinderungen unterscheiden sich in der Inanspruchnahme zahnmedizinischer Versorgung: Während 21,5 % der Personen mit Beeinträchtigungen und Behinderungen im Jahr vor der Befragung keine zahnmedizinischen Leistungen in Anspruch genommen hatten, waren es bei den Personen ohne Beeinträchtigungen und Behinderungen mit 18,4 % etwas weniger. Dieser Unterschied ist statistisch signifikant, wie das dazugehörige univariate log-Poisson-Regressionsmodell zeigt (univariate PR: 1,2, *p* = 0,002, Ergebnisse nicht gezeigt).

**Figure 1:**
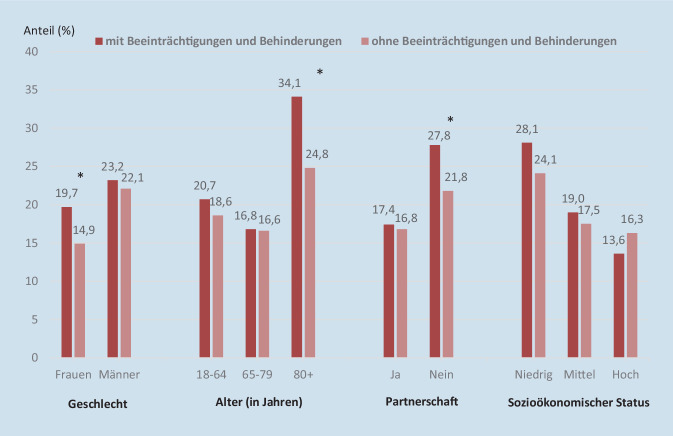

Der Anteil der Personen, die im Jahr vor der Befragung keine zahnmedizinischen Leistungen in Anspruch genommen hatten, variiert bei Erwachsenen mit und ohne Beeinträchtigungen und Behinderungen nach den herangezogenen soziodemografischen Merkmalen (Abb. 1): Frauen mit Beeinträchtigungen und Behinderungen (19,7 %) nahmen im Vergleich zu Frauen ohne Beeinträchtigungen und Behinderungen (14,9 %) häufiger keine zahnmedizinischen Leistungen in Anspruch (univariate PR: 1,3; *p* < 0,001). Zwischen Männern mit und ohne Beeinträchtigungen und Behinderungen zeigten sich keine signifikanten Unterschiede in der Inanspruchnahme. Bei Männern, und zwar unabhängig vom Vorliegen einer Beeinträchtigung oder Behinderung, lag die Nichtinanspruchnahme höher als bei Frauen mit und vor allem ohne Beeinträchtigungen und Behinderungen. Sie lag jeweils bei über 20 %.

Darüber hinaus zeigte sich ein deutlicher Unterschied in der Inanspruchnahme zahnmedizinischer Versorgung zwischen Personen mit und ohne Beeinträchtigungen und Behinderungen, wenn diese mit einer Partnerin bzw. einem Partner im Haushalt leben (27,8 % bzw. 21,8 %, univariate PR: 1,3, *p* < 0,001). Der stärkste Unterschied zeigte sich allerdings im hohen Alter: Während mehr als ein Drittel (34,1 %) der 80-jährigen und älteren Personen mit Beeinträchtigungen und Behinderungen im Jahr vor der Befragung keine zahnmedizinische Praxis aufgesucht hatte, war es bei den Gleichaltrigen ohne Beeinträchtigungen und Behinderungen mit 24,8 % nur rund ein Viertel (univariate PR: 1,4, *p* = 0,012). Unterschiede in der Inanspruchnahme zahnmedizinischer Versorgung zwischen Personen mit und ohne Beeinträchtigungen und Behinderungen nach SES waren nicht festzustellen (Abb. 1).

Der Zusammenhang zwischen dem Vorliegen von Beeinträchtigungen und Behinderungen und der Inanspruchnahme zahnmedizinischer Leistungen, der sich univariat zeigte, war nach statistisch wechselseitiger Kontrolle aller Merkmale nicht mehr signifikant (multivariates Modell, Tab. 2). Männliches Geschlecht, ein hohes Alter (80 Jahre und älter), keine Partnerin bzw. Partner im Haushalt sowie die Zugehörigkeit zur niedrigen sozioökonomischen Statusgruppe erklären den Zusammenhang (*p* jeweils > 0,001).

**Table 2:** 

	PR (95 %-KI)^a^	*p*-Wert^a^
*Beeinträchtigungen und Behinderungen*		
Nein	Ref.	–
Ja	1,0 (0,9–1,1)	0,730
Geschlecht		
Frauen	Ref.	–
Männer	1,5 (1,3–1,7)	< 0,001
*Altersgruppe*		
18–64 Jahre	Ref.	–
65–79 Jahre	0,9 (0,8–1,0)	0,034
80 Jahre und älter	1,5 (1,3–1,7)	< 0,001
*Partnerschaft*		
Ja	Ref.	–
Nein	1,3 (1,2–1,4)	< 0,001
*Sozioökonomischer Status*		
Niedrig	1,5 (1,3–1,6)	< 0,001
Mittel	1,1 (1,0–1,2)	0,013
Hoch	Ref.	–

*PR* Prevalence Ratio, *KI* Konfidenzintervall, *Ref.* Referenzgruppe

^a^Ergebnisse aus multivariaten log-Poisson-Regressionen

## Diskussion

Ziel dieser Arbeit war es, auf Datenbasis einer für Deutschland repräsentativen Stichprobe mögliche Unterschiede in der Inanspruchnahme zahnmedizinischer Versorgung bei Erwachsenen mit und ohne Beeinträchtigungen und Behinderungen nach soziodemografischen Merkmalen zu ermitteln. Durch die vorliegende Publikation können diesbezüglich nun erstmals Aussagen getroffen werden. Den Ergebnissen aus GEDA 2014/2015-EHIS zufolge geht das Vorliegen von Beeinträchtigungen und Behinderungen mit einer geringeren Inanspruchnahme zahnmedizinischer Leistungen einher. Dieser Zusammenhang bleibt aber nicht bestehen, wenn zusätzlich weitere Faktoren im Modell berücksichtigt werden, die mit einer geringeren Inanspruchnahme [26] assoziiert sind: Männliches Geschlecht, ein hohes Alter, das Leben ohne Partnerin bzw. Partner im Haushalt und ein niedriger SES erklären den untersuchten Zusammenhang.

Nach Kenntnis des Autor:innenteams gibt es bisher keine Studie aus Deutschland, die die Inanspruchnahme zahnmedizinischer Versorgung von Erwachsenen mit und ohne Beeinträchtigungen und Behinderungen analysiert hat. Eine deutsche Studie zur allgemeinen Inanspruchnahme ärztlicher Leistungen von Menschen mit und ohne Behinderungen (ab 18 Jahre), in der ebenfalls Daten aus GEDA untersucht wurden, konnte zeigen, dass Erwachsene mit Behinderungen – sowohl mit einem Grad der Behinderung von unter als auch über 50 – häufiger eine zahnmedizinische Praxis aufsuchen als Personen ohne Behinderungen [16]. Dieser Unterschied war auch nach Kontrolle für Geschlecht, Alter, Wohnort (Land/Stadt), selbsteingeschätzte Gesundheit und dem Vorliegen chronischer Erkrankungen festzustellen. Allerdings war auch in dieser Arbeit der Unterschied in der Inanspruchnahme zwischen den Gruppen relativ gering (ohne Behinderung: 80,8 %, GdB < 50: 85,5 %, GdB ≥ 50: 82,0 %; [16]). Eine andere deutsche Studie zur gesundheitlichen Versorgung von Menschen mit geistiger Behinderung in Werkstätten (ab 18 Jahre, 68 % der Teilnehmenden hatten einen GdB von 100) kam zu dem Ergebnis, dass Erwachsene mit geistiger Behinderung gleich häufig zahnärztliche Kontrolluntersuchungen in Anspruch nehmen wie Personen aus der Allgemeinbevölkerung (82,1 % bzw. 79,4 %; verglichen wurden die Daten mit Daten der Studie zur Gesundheit Erwachsener in Deutschland (DEGS1, 2008–2011) des RKI; [10]). Die Daten der Fünften Deutschen Mundgesundheitsstudie (DMS V) des Instituts der Deutschen Zahnärzte (IDZ) weisen ebenfalls darauf hin, dass sich Erwachsene mit und ohne Schwerbehinderung (65–74 Jahre) nicht in der zahnmedizinischen Inanspruchnahme unterscheiden (kontrollorientierte Inanspruchnahme: 89,1 % bzw. 89,6 %, beschwerdeorientierte Inanspruchnahme: 10,9 % bzw. 10,4 %; [15]). Dass nur geringe Unterschiede bestehen, zeigt sich bereits im Kindes- und Jugendalter: Rund drei Viertel der Kinder und Jugendlichen mit und ohne Behinderungen gehen 2‑mal im Jahr zur zahnärztlichen Kontrolluntersuchung (77,2 % bzw. 74,6 %; [27]). Internationale Studien liefern ein inkonsistentes Bild und verweisen auf geringe bis deutliche Unterschiede in der Inanspruchnahme zahnmedizinischer Leistungen zuungunsten von Erwachsenen mit Beeinträchtigungen und Behinderungen [28–32]. Ein direkter Vergleich ist aufgrund der unterschiedlichen Gesundheits- und Sozialsysteme aber nicht möglich.

Ein weiteres Ergebnis dieser Arbeit ist, dass sich die Inanspruchnahme zahnmedizinischer Leistungen zwischen Personen mit und ohne Beeinträchtigungen und Behinderungen in ausgewählten Untergruppen unterscheidet. Aus der wissenschaftlichen Literatur ist bekannt, dass Frauen mit Beeinträchtigungen und Behinderungen stärker von gesundheitlicher Ungleichheit betroffen sind [33]. Sie sind oft mehrfach benachteiligt, aufgrund ihrer Behinderung und ihres Geschlechts [5]. Dies spiegelt sich in den vorliegenden Ergebnissen nur zum Teil wider: Bei Frauen mit Beeinträchtigungen und Behinderungen ist die Nichtinanspruchnahme zahnmedizinischer Leistungen höher als bei Frauen ohne Beeinträchtigungen und Behinderungen. Es wird aber auch deutlich, dass die Inanspruchnahme zahnmedizinischer Versorgung bei Frauen höher ist als bei Männern – dieser Befund gilt allgemein für die Inanspruchnahme gesundheitlicher Leistungen [33]. Zudem zeigen sich Unterschiede in der Inanspruchnahme zahnmedizinischer Leistungen zwischen Personen mit und ohne Beeinträchtigungen und Behinderungen im hohen Alter. Weiterführende Analysen sprechen dafür, dass eine Interaktion zwischen Alter und Geschlecht besteht (*p* < 0,001). So weisen 80-jährige und ältere Frauen mit Beeinträchtigungen und Behinderungen eine um 10 Prozentpunkte höhere Nichtinanspruchnahme zahnmedizinischer Versorgung auf als gleichaltrige Frauen ohne Beeinträchtigungen und Behinderungen (37,7 % bzw. 28,8 %), während in den jüngeren Altersgruppen (18–64 Jahre und 65–79 Jahre) keine Unterschiede in der Inanspruchnahme zwischen Frauen mit und ohne Beeinträchtigungen und Behinderungen zu beobachten sind (die Nichtinanspruchnahme beträgt hier in allen Untergruppen etwa 15 %; die Ergebnisse dieser Sensitivitätsanalyse sind nicht gezeigt). In der Studie zur gesundheitlichen Versorgung von Menschen mit geistiger Behinderung in Werkstätten konnte gezeigt werden, dass Erwachsene mit geistiger Behinderung aus Wohnheimen zahnärztliche Kontrolluntersuchungen häufiger wahrnehmen als Personen mit geistiger Behinderung, die alleine wohnen [10]. Dieses Ergebnis steht im Einklang mit dem vorliegenden Befund, dass das Leben ohne Partnerin bzw. Partner mit einer geringeren Inanspruchnahme zahnmedizinischer Versorgung einhergeht. Eine Interaktion zwischen Partnerschaft und Alter bzw. Partnerschaft und Geschlecht besteht den Ergebnissen zufolge nicht.

### Stärken und Limitationen dieser Arbeit

Nach Kenntnis des Autor:innenteams ist der vorliegende Beitrag der erste, der die Inanspruchnahme zahnmedizinischer Leistungen von Erwachsenen im Vergleich zu Erwachsenen ohne Beeinträchtigungen und Behinderungen basierend auf bundesweiten, repräsentativen Befragungsdaten untersucht. Die Analysen wurden mit GEDA 2014/2015-EHIS durchgeführt, einem Datensatz, der Teilnehmende vom jungen bis ins hohe Erwachsenenalter umfasst. Die hohe Teilnehmendenzahl ermöglicht Stratifizierungen nach verschiedenen soziodemografischen Merkmalen und dadurch eine detaillierte Betrachtung der Inanspruchnahme in ausgewählten Untergruppen. Allerdings ist limitierend auf das Alter der Datenquelle (2014/2015) hinzuweisen. Im RKI-Gesundheitsmonitoring stehen bislang aber keine neueren Daten zur Verfügung, mit denen das Vorliegen von Beeinträchtigungen und Behinderungen kombiniert ausgewertet werden kann [21]. Auch ist eine Teilnahme an Surveys, die sich an die Allgemeinbevölkerung richten, für Personen, die von gesundheitlichen Einschränkungen betroffen sind, manchmal nur schwer oder gar nicht realisierbar, z. B. das Ausfüllen von Papier- oder Onlinefragebögen durch Menschen mit Sehbehinderung. Daraus können eine selektive Nichtteilnahme und infolgedessen eine Unterrepräsentierung und Verzerrung der Ergebnisse (Selektionsbias) resultieren [34]. Zudem wurden Menschen, die in Wohneinrichtungen oder Pflegeheimen leben, nicht in die Erhebung einbezogen. Dies unterstreicht die hohe Bedeutung der wenigen Primärdatenstudien, die in derartigen Einrichtungen durchgeführt wurden [7, 9, 10].

Für die Operationalisierung von Beeinträchtigungen und Behinderungen gibt es zudem unterschiedliche Möglichkeiten. In diesem Beitrag wurde, im Einklang mit vorangegangenen Auswertungen von GEDA 2014/2015-EHIS, die Definition aus dem Zweiten Teilhabebericht [20] zugrunde gelegt. Der aktuellere Dritte Teilhabebericht [3] nutzt eine erweiterte Definition und schließt zusätzlich Personen mit leichten Behinderungen ein. In anderen Untersuchungen wurden nur Personen mit amtlich anerkannten Behinderungen einbezogen [15, 16, 27]. Eine belastbare, wenn auch aufwendige Lösung stellt der Teilhabesurvey dar, in dem die selbsteingeschätzte Behinderung der Teilnehmenden erhoben und für die Analysen verwendet wird [37].

Darüber hinaus ist zu berücksichtigen, dass aus der vorliegenden Arbeit keine Rückschlüsse auf die Mundgesundheit und auf die Gründe für die Inanspruchnahme zahnmedizinischer Versorgung möglich sind. Die in GEDA befragten Personen können präventive, aber auch therapeutische Leistungen in Anspruch genommen haben. Zudem können Selbstangaben zur Inanspruchnahme gesundheitlicher Leistungen mit Erinnerungslücken (Recall Bias) verbunden sein [35]; dies betrifft aber eher die Anzahl der Kontakte als die Frage, ob niedergelassene Ärztinnen und Ärzte überhaupt in Anspruch genommen wurden. Außerdem ist ein Recall Bias wahrscheinlicher, wenn ein längerer Zeitraum als die letzten 12 Monate erfasst wird [36].

### Ansatzpunkte zur Förderung der Mundgesundheit bei Menschen mit Beeinträchtigungen und Behinderungen

Menschen mit Beeinträchtigungen und Behinderungen sind eine sehr heterogene Gruppe mit ganz unterschiedlichen gesundheitlichen Situationen und Bedarfen [20]. Aufgrund ihres im Mittel schlechteren Gesundheitszustands weisen sie eine höhere Inanspruchnahme ambulanter und stationärer Versorgung auf als Menschen ohne Beeinträchtigungen und Behinderungen [10, 16, 21, 33]. Auch der Mundgesundheitszustand ist bei ihnen im Mittel schlechter: Im Vergleich zu Personen ohne Beeinträchtigungen und Behinderungen sind sie häufiger von Karies, Parodontitis und Zahnverlust betroffen [6, 15, 38]. Mit Blick auf die Inanspruchnahme zahnmedizinischer Leistungen vermitteln die vorliegenden Analysen das Bild, dass sich Personen mit und ohne Beeinträchtigungen und Behinderungen darin kaum voneinander unterscheiden. Allerdings konnte in GEDA 2014/2015-EHIS nicht zwischen den verschiedenen Arten von Beeinträchtigung und Behinderung unterschieden werden. Auch die Art der zahnmedizinischen Untersuchung oder Behandlung wurde nicht erfasst. Studien zeigen, dass bestimmte Gruppen von Menschen mit Behinderungen aufwendigere medizinische Untersuchungen, wie z. B. die Früherkennungsuntersuchungen für Dickdarm‑, Gebärmutterhals- oder Prostatakrebs, seltener wahrnehmen [10, 31]. Auch zahnärztliche Untersuchungen und Behandlungen, wie etwa die Parodontalbehandlung, sind bei Menschen mit Behinderungen mitunter schwieriger durchzuführen [39].

Vor diesem Hintergrund ist zu überlegen, wie die zahnmedizinische Versorgung dieser sehr heterogenen Gruppe weiter verbessert werden kann. Entsprechende Vorschläge wurden von Bundeszahnärztekammer (BZÄK) und Kassenzahnärztlicher Bundesvereinigung (KZBV) für Menschen mit Behinderungen vor Kurzem formuliert [40, 41]. Ein wichtiger Aspekt für eine bedarfsgerechte Versorgung ist die Barrierefreiheit zahnärztlicher Praxen [42]. Eine Übersicht findet sich auf der Webseite der BZÄK [43]. Die Kassenzahnärztlichen Vereinigungen (KZVen) Berlin und Mecklenburg-Vorpommern bieten auf ihren Websites auch einen zahnärztlichen Praxisführer für Menschen mit Behinderungen an [44, 45]. Zudem ist die Aufnahme des Themas Versorgung von Menschen mit Behinderungen in die zahnärztliche Weiterbildung von großer Bedeutung. Zwar werden in der zahnärztlichen Approbationsordnung, die seit 2020 gültig ist, coronabedingt aber ausgesetzt war und erst seit 2021 umgesetzt wird, erstmals Inhalte zu zahnmedizinisch relevanten Besonderheiten bei der Behandlung spezieller Gruppen von Patientinnen und Patienten, wie etwa Menschen mit Behinderungen, im Rahmen einer der staatlichen Abschlussprüfungen im Studiengang Zahn‑, Mund- und Kieferheilkunde als prüfungsrelevant angesehen [46]. Allerdings gibt es bislang keine einheitlichen Mindestinhalte für ein entsprechendes Curriculum [6]. Ferner ist eine interdisziplinäre Zusammenarbeit der Zahnärztinnen und Zahnärzte mit Hausärztinnen und Hausärzten sowie Pflegediensten sinnvoll, da diese von Menschen mit Beeinträchtigungen und Behinderungen sehr häufig in Anspruch genommen werden [10, 16, 21]. Bei einem eingeschränkten Zugang zu (zahn-)ärztlichen Versorgungsleistungen können aufsuchende Mundgesundheitsdienste unterstützen, z. B. durch die gruppenprophylaktische Betreuung in integrativen Einrichtungen [6]. Abschließend ist anzumerken, dass durch regelmäßige zahnärztliche Kontrolluntersuchungen Erkrankungen der Zähne und des Mundraums frühzeitig erkannt und geeignete Maßnahmen eingeleitet werden können [47]. Somit ist der zahnmedizinischen Prävention für Menschen mit Beeinträchtigungen und Behinderungen eine ganz besondere Bedeutung zuzuschreiben.

## Fazit

In der Inanspruchnahme zahnmedizinischer Versorgung sind kaum Unterschiede zwischen Menschen mit und ohne Beeinträchtigungen und Behinderungen festzustellen. Aufgrund ihrer im Mittel schlechteren Mundgesundheit ist dennoch zu überlegen, wie die zahnmedizinische Versorgung dieser sehr heterogenen Gruppe weiter verbessert werden kann. Die vorliegenden Auswertungen zeigen Versorgungsbedarfe und Präventionspotenziale auf. Vor dem Hintergrund der älteren sowie lückenhaften Datenlage besteht weiterhin Forschungsbedarf zur Mundgesundheit von Menschen mit Beeinträchtigungen und Behinderungen in Deutschland.

## References

[1] Statistisches Bundesamt (2021) Öffentliche Sozialleistungen – Lebenslagen der behinderten Menschen. Ergebnisse des Mikrozensus 2019. https://www.destatis.de/DE/Themen/Gesellschaft-Umwelt/Gesundheit/Behinderte-Menschen/Publikationen/Downloads-Behinderte-Menschen/lebenslagen-behinderter-menschen-5122123199004.pdf?__blob=publicationFile. Zugegriffen: 7. März 2023

[2] Statistisches Bundesamt (2022) Schwerbehinderte Menschen mit Ausweis (absolut und je 100.000 Einwohner). Statistik der schwerbehinderten Menschen. www.gbe-bund.de. Zugegriffen: 7. März 2023

[3] Bundesministerium für Arbeit und Soziales (2021). Dritter Teilhabebericht der Bundesregierung über die Lebenslagen von Menschen mit Beeinträchtigungen. Teilhabe – Beeinträchtigung – Behinderung.

[4] Bundesministerium für Arbeit und Soziales (2016). Unser Weg in eine inklusive Gesellschaft. Nationaler Aktionsplan 2.0 der Bundesregierung zur UN-Behindertenrechtskonvention (UN-BRK).

[5] Vereinte Nationen (2006) Die UN-Behindertenrechtskonvention. Übereinkommen der Vereinten Nationen über die Rechte von Menschen mit Behinderungen. www.behindertenbeauftragte.de/SharedDocs/Publikationen/UN_Konvention_deutsch.pdf?__blob=publicationFile&v=2. Zugegriffen: 7. März 2023

[6] Schulte AG, Schmidt P (2021). Mundgesundheit bei Menschen mit Behinderung in Deutschland – eine Literaturübersicht. Bundesgesundheitsblatt Gesundheitsforschung Gesundheitsschutz.

[7] Schulte AG, Freyer K, Bissar A (2013). Caries experience and treatment need in adults with intellectual disabilities in two German regions. Community Dent Health.

[8] Schulte AG, Kaschke I, Bissar A (2011). Mundgesundheit erwachsener Athleten mit geistiger Behinderung. Gesundheitswesen.

[9] Schmidt P, Egermann M, Sauerland C, Schulte AG (2021). Caries experience of adults with intellectual disability in the western part of Germany. J Clin Med.

[10] Wellkamp R, De Cruppé W, Schwalen S, Geraedts M (2022). Inanspruchnahme der gesundheitlichen Versorgung von Menschen mit geistiger Behinderung, eine Querschnittstudie in 3 Werkstätten. Gesundheitswesen.

[11] Bundeszahnärztekammer (BZÄK) Gesunde Zähne, gesunder Körper – gesunder Körper, gesunde Zähne. www.bzaek.de/fileadmin/PDFs/bv/GesundeZaehne_Koerper_CP.pdf. Zugegriffen: 7. Juli 2022

[12] Casanova L, Hughes FJ, Preshaw PM (2014). Diabetes and periodontal disease: a two-way relationship. Br Dent J.

[13] Marchetti E, Monaco A, Procaccini L (2012). Periodontal disease: the influence of metabolic syndrome. Nutr Metab.

[14] Gomes-Filho IS, Passos JS, Seixas Da Cruz S (2010) Respiratory disease and the role of oral bacteria. J Oral Microbiol 2:5811 - 10.3402/jom.v2i0.5811 10.3402/jom.v2i0.5811 PMC3084574 21523216

[15] Institut Der Deutschen Zahnärzte (Idz) (2016). Fünfte Deutsche Mundgesundheitsstudie (DMS V).

[16] Wetzel LD, Rathmann K (2020). Inanspruchnahme und wahrgenommene Barrieren des Gesundheitswesens bei Menschen mit Behinderung in Deutschland: Ergebnisse des GEDA 2014/2015-EHIS-Survey. Präv Gesundheitsf.

[17] Robert Koch-Institut (2017). Fragebogen zur Studie „Gesundheit in Deutschland aktuell“: GEDA 2014/2015-EHIS. J Health Monit.

[18] European Commission, Eurostat (2013). European Health Interview Survey (EHIS wave 2). Methodological manual.

[19] Krause L, Seeling S, Starker A (2021). Selbstwahrgenommene Mundgesundheit und assoziierte Faktoren bei Erwachsenen in Deutschland. Ergebnisse aus GEDA 2019/2020-EHIS. Bundesgesundheitsblatt Gesundheitsforschung Gesundheitsschutz.

[20] Bundesministerium für Arbeit und Soziales (2016). Zweiter Teilhabebericht der Bundesregierung über die Lebenslagen von Menschen mit Beeinträchtigungen. Teilhabe – Beeinträchtigung – Behinderung.

[21] Prütz F, Krause L (2022). Gesundheit von Menschen mit Beeinträchtigungen und Behinderungen in Deutschland – Ausgewählte Indikatoren aus der Studie GEDA 2014/2015-EHIS. J Health Monit.

[22] Lampert T, Kroll LE, Müters S, Stolzenberg H (2013). Messung des soziookonomischen Status in der Studie „Gesundheit in Deutschland aktuell“ (GEDA). Bundesgesundheitsblatt Gesundheitsforschung Gesundheitsschutz.

[23] Eurostat (2016) Internationale Standardklassifikation für das Bildungswesen (ISCED). http://ec.europa.eu/eurostat/statistics-explained/index.php/Glossary:International_standard_classification_of_education_%28ISCED%29/de. Zugegriffen: 7. März 2023

[24] Saß A-C, Lange C, Finger JD (2017). „Gesundheit in Deutschland aktuell“ – Neue Daten für Deutschland und Europa, Hintergrund und Studienmethodik von GEDA 2014/2015-EHIS. J Health Monit.

[25] Lange C, Finger JD, Allen J (2017). Implementation of the European health interview survey (EHIS) into the German health update (GEDA). Arch Public Health.

[26] Krause L, Frenzel Baudisch N, Bartig S, Kuntz B (2020). Inanspruchnahme einer Zahnvorsorgeuntersuchung durch Erwachsene. Ergebnisse der GEDA-Studie 2009, 2010, 2012. Dtsch Zahnärztl Z.

[27] Krause L, Seeling S, Prütz F, Wager J (2022). Toothache, tooth brushing frequency and dental check-ups in children and adolescents with and without disabilities. J Health Monit.

[28] Patel N, Fils-Aime R, Li CH, Lin M, Robison V (2021). Prevalence of past-year dental visit among US adults aged 50 years or older, with selected chronic diseases, 2018. Prev Chronic Dis.

[29] Leroy R, Declerck D (2013). Oral health-care utilization in adults with disabilities in Belgium. Eur J Oral Sci.

[30] Lim HJ, Im A, Cho HA (2019). The association between visual impairment and dental care utilization in the Korean elderly. Arch Gerontol Geriatr.

[31] Havercamp SM, Scandlin D, Roth M (2004). Health disparities among adults with developmental disabilities, adults with other disabilities, and adults not reporting disability in North Carolina. Public Health Rep.

[32] Chavis SE, Macek M (2022). Impact of disability diagnosis on dental care use for adults in the United States: Status matters. J Am Dent Assoc.

[33] Robert Koch-Institut (2020). Gesundheitliche Lage der Frauen in Deutschland. Gesundheit von Frauen mit Behinderungen (Kapitel 9). Gesundheitsberichterstattung des Bundes.

[34] Ohlmeier C, Frick J, Prütz F (2014). Nutzungsmöglichkeiten von Routinedaten der Gesetzlichen Krankenversicherung in der Gesundheitsberichterstattung des Bundes. Bundesgesundheitsblatt Gesundheitsforschung Gesundheitsschutz.

[35] Hessel A, Gunzelmann T, Geyer M, Brahler E (2000). Inanspruchnahme medizinischer Leistungen und Medikamenteneinnahme bei über 60jährigen in Deutschland – gesundheitliche, sozialstrukturelle, sozio-demographische und subjektive Faktoren. Z Gerontol Geriat.

[36] Bhandari A, Wagner T (2006). Self-reported utilization of health care services: improving measurement and accuracy. Med Care Res Rev.

[37] Schröder H, Steinwede J, Schäfers M, Kersting A, Harand J (2017) 1. Zwischenbericht – Repräsentativbefragung zur Teilhabe von Menschen mit Behinderungen. https://www.bmas.de/SharedDocs/Downloads/DE/Publikationen/Forschungsberichte/fb-492-repraesentativbefragung-behinderung.pdf?__blob=publicationFile&v=1. Zugegriffen: 22. Nov. 2022

[38] Griffin SO, Barker LK, Griffin PM, Cleveland JL, Kohn W (2009). Oral health needs among adults in the United States with chronic diseases. J Am Dent Assoc.

[39] Kassenzahnärztliche Bundesvereinigung (KZBV), Bundeszahnärztekammer (BZÄK) (2010) Mundgesund trotz Handicap und hohem Alter. Konzept zur vertragszahnärztlichen Versorgung von Pflegebedürftigen und Menschen mit Behinderungen. https://www.kzbv.de/konzept-mundgesund.download.08b5be6f8cc0fdedac2297fa1219037b.pdf. Zugegriffen: 30. Sept. 2022

[40] Kassenärztliche Bundesvereinigung (Kzbv) (2022) https://www.kzbv.de/verbesserung-der-zahnaerztlichen-versorgung-von.1620.de.html. Zugegriffen: 30. Sept. 2022

[41] Bundeszahnärztekammer (Bzäk) (2022) Zahnmedizin für Menschen mit Behinderung und medizinischem Unterstützungsbedarf. Presseinformation. https://www.bzaek.de/presse/presseinformationen/presseinformation/zahnmedizin-fuer-menschen-mit-behinderung-medizinischem-unterstuetzungsbedarf-hochbetagte-und-pflegebeduerftige.html. Zugegriffen: 30. Sept. 2022

[42] Hansen C, Curl C, Geddis-Regan A (2021). Barriers to the provision of oral health care for people with disabilities. BDJ Pract.

[43] Bundeszahnärztekammer (BZÄK) (2022) Barrierefreie Zahnarztpraxen. https://www.bzaek.de/praevention/alters-und-behindertenzahnmedizin/barrierefreie-zahnarztpraxen.html. Zugegriffen: 7. März 2023

[44] Zahnärztekammer Berlin (2012) Zahnärztlicher Praxisführer für Menschen mit Behinderungen und geriatrische Patienten. https://www.zaek-berlin.de/fileadmin/dokumente/patienten/ratgeber/ZAEK_Praxisfuehrer_2016.pdf. Zugegriffen: 7. Nov. 2022

[45] Zahnärztekammer Mecklenburg-Vorpommern (2022) Zahnärztlicher Praxisführer für Patienten mit Behinderungen und geriatrische Patienten. https://www.zaekmv.de/fileadmin/Redaktion/Downloads_AuB/Praxisfuehrer.pdf. Zugegriffen: 7. Nov. 2022

[46] Bundesgesetzblatt (2019) Verordnung zur Neuregelung der zahnärztlichen Ausbildung. http://www.bgbl.de/xaver/bgbl/start.xav?startbk=Bundesanzeiger_BGBl&jumpTo=bgbl119s0933.pdf. Zugegriffen: 19. Dez. 2022

[47] Deutsche Gesellschaft für Zahn‑, Mund- und Kieferheilkunde e. V. (DGZMK), Deutsche Gesellschaft für Zahnerhaltung e. V. (DGZ) (2016) S2k-Leitlinie: Kariesprophylaxe bei bleibenden Zähnen – grundlegende Empfehlungen. AWMF-Registernummer 083-021. https://www.awmf.org/leitlinien/detail/ll/083-021.html. Zugegriffen: 6. Juli 2022

